# Awareness, Knowledge, and Perceptions of COVID-19 Precautions Among Employees of Al-Imam Abdulrahman Al Faisal Hospital in Riyadh, KSA

**DOI:** 10.7759/cureus.25918

**Published:** 2022-06-13

**Authors:** Ateeg M Alqarni, Mohammed Bajahzer, Mohammed Asseri, Ayman S Alahmari, Sarah Alkhaldi, Farkad Bantun, Abdullah H Alhamoud

**Affiliations:** 1 Department of Urology, Al-Imam Abdulrahman Al Faisal Hospital, First Cluster, Ministry of Health, Riyadh, SAU; 2 Department of Clinical Nutrition, Applied Medical Sciences, Jazan University, Jazan, SAU; 3 Department of Emergency Medicine, Al-Imam Abdulrahman Al Faisal Hospital, First Cluster, Ministry of Health, Riyadh, SAU; 4 Department of Laboratory, Al-Imam Abdulrahman Al Faisal Hospital, First Cluster, Ministry of Health, Riyadh, SAU; 5 Department of Microbiology, Faculty of Medicine, Umm Al-Qura University, Makkah, SAU; 6 General Pediatrics, King Fahad Central Hospital, Jazan, SAU

**Keywords:** al-imam abdulrahman al-faisal hospital, hcp, covid-19, perception, knowledge

## Abstract

Background

The global pandemic of coronavirus disease-19 (COVID-19) was announced by the World Health Organization (WHO) in early 2020. The consequences of the pandemic were vast, where healthcare systems, education, and the economy of many countries were greatly affected. As such, extraordinary precautions and measures were implemented to the public as well as to the healthcare systems in order to counter the spread of the disease. However, the success of these measures depends largely on the individual's adherence to them as well as their awareness about COVID-19. Indeed, healthcare workers and their non-medical co-workers play a crucial role in that, as they are considered the front line in fighting the infection.

Objectives

To assess the knowledge, awareness and perceptions of the healthcare workers (HCWs) regarding COVID-19 in Al-Imam Abdulrahman Al Faisal Hospital in Riyadh, Saudi Arabia.

Methods

Using a cross-sectional study design, a previously validated questionnaire was used as an online survey to assess the knowledge, awareness and perceptions (KAP) of HCWs regarding COVID-19. The targeted population of this study was all the healthcare workers in Al-Imam Abdulrahman Al Faisal Hospital, including their non-medical co-workers.

Results

The study included 274 respondents consisting of 53.65% males and 46.45% females with an average age between 30-39 years. The majority of the respondents were paramedics with a percentage of 30.66%. The governmental references were the main source of information regarding COVID-19 for 69% of the respondents. Questions with accurate responses that exceeded 90% were about the COVID-19 complications, transmission, and measures to reduce its transmission. In this study, overall knowledge was significantly associated with the gender of the participants (P=0.01).

Conclusion

There was a good level of knowledge and perception in health care professionals and co-workers regarding SARS-CoV-2.

## Introduction

In December 2019, scientists in Wuhan, China, separated a new RNA virus called severe acute respiratory syndrome coronavirus 2 (SARS-CoV-2) within pneumonia cases. The infection induced by this virus is named coronavirus disease-19 (COVID-19) [1]. The rapid outbreak of COVID-19 affected about 219 countries worldwide resulting in more than 180 million COVID-19 cases and over 3 million deaths [2]. In Saudi Arabia, 480,000 COVID-19 cases and 7,000 deaths as of the 19th of June 2021 were reported. Therefore, the outbreak of COVID-19 is a major priority for public health worldwide [3]. COVID-19 spreads mainly through close contact with respiratory droplets of an infected person or surfaces contaminated with the virus. The incubation period of the virus lasts up to 14 days in symptomatically or asymptomatically COVID-19 patients [4]. The common symptoms of COVID-19 are fever, fatigue, shortness of breath, dry cough, dyspnea, and decreased white blood cells [5]. Further, headache, diarrhea, anorexia, abdominal pain, sore throat, and rhinorrhea are less common symptoms of COVID-19 [6]. The effect of the COVID-19 pandemic has been vast to the extent that it has wreaked havoc on the whole global community in addition to most of the health care, educational, and economic national systems [7]. Right after the announcement of the COVID-19 outbreak, the Saudi Ministry of Health (MOH) took serious precautionary measures to promote public awareness and knowledge to limit the spread of COVID-19. Information about COVID-19 symptoms, ways of transmission, and preventive measures were the focus of all the Saudi national press and media to prevent the spread of the virus. Further public legislation, such as social distancing, face covering, and panning social gatherings in addition to providing free face masks and hand sanitizing products to the public helped greatly limit the chain of the infection [8]. Healthcare workers (HCWs) interact directly with patients, thereby at a high risk of being exposed to COVID-19. Therefore, WHO and the Centre for Disease Control and Prevention (CDC) have targeted the HCWs with various COVID-19 educational, training, and recommendation guidelines that have been published online [9, 10]. Nonetheless, the cases of COVID-19 among the HCWs are considerably high, implying the possibility of COVID-19 knowledge or practice gaps [11-13]. The level of COVID-19 knowledge among the HCWs has been examined in various studies that would deepen our understanding to develop further solutions [14-17]. Nonetheless, there is still a gap in the literature on the knowledge and perceptions of HCWs in Saudi Arabia. Thus, we aimed to assess the knowledge and perceptions of COVID-19 among the HCWs at Al-Imam Abdulrahman Al Faisal Hospital in Riyadh, Saudi Arabia. Such a study can provide further insight on the status of the HCWs' knowledge of COVID-19 and if more efforts are consequently required.

## Materials and methods

This cross-sectional study was conducted using a web-based self-reported questionnaire to assess the level of HCW's knowledge and perceptions regarding COVID-19 in Al-Imam Abdulrahman Al Faisal Hospital in Riyadh, Saudi Arabia. The study took place between June and August 2020 where we targeted all the employees of the hospital who were on duty during the period of the study - around 900 employees. A total of 274 subjects agreed to participate and complete the questionnaire, which was estimated previously as the appropriate sample size for this study with confidence limits of 95% and 5% precision. The questionnaire was composed of 13 multiple choice questions that were divided into two sections: the first section inquired about demographic information and the second section inquired about COVID-19 knowledge and practice. The level of knowledge assessment was based on the correct responses to seven of the questions that were relevant to COVID-19 knowledge [18].

Ethical considerations

This study was reviewed and got approval from the institutional review board in Al-Imam Abdulrahman Al-Faisal Hospital, AIAAH 2020-P (reference number is AIAAH/2020/AIAAHC/001-1). Informed consent was obtained from all the participations. The consent details and forms were displayed to all participants for approval before directing them to the questionnaire. The questionnaire was answered by all the participants anonymously throughout the study period to maintain the confidentiality of participants' data.

Statistical analysis

The acquired data were analyzed using SPSS version 22 (IBM Corp., Armonk, NY). The characteristics of participants and their answers to the questionnaire were quantified in frequencies or/and in relative percentages. Categorical variables were compared using the chi-square test or Fisher’s exact test if the sample distribution assumption (more than 20% of cells in the contingency table have expected frequencies <5) was not met. The significance level was considered at a p-value of ≤ 0.05.

The overall knowledge of the HCWs was considered on a relative scale of the correct answers to the overall number of the questions relevant to COVID-19 knowledge. The cut-off level for sufficient knowledge was considered at ≥ 60%.

## Results

This study included 274 participants, which comprised 53.65% males and 46.35% females. The dominant age group among them was 30-39 years (47.45%), and the paramedics represented the majority (30.66%) of participants' profession. All the HCWs, except for one, indicated that they heard about SARS-CoV-2. Despite that, only 70% have attended or participated in educational sessions about the disease (Table 1).

**Table 1:** Characteristics of the participants

Sum Participants (n) = 274	Number	(%)
Gender		
Male	147	(53.65)
Female	127	(46.35)
Age group		
< 30	67	(24.45)
30 - 39	130	(47.45)
40 - 49	56	(20.44)
50 - 59	16	(5.84)
> 59	5	(1.82)
Profession		
Physician	65	(23.72)
Nurse	62	(22.63)
Paramedics	84	(30.66)
Hospital administrator	63	(22.99)
Heard about (SARS-CoV-2)?		
Yes	273	(99.64)
No	1	(0.36)
Attended any lectures/discussions about (SARS-CoV-2) disease?		
Yes	192	(70.07)
No	82	(29.93)

**Figure 1:**
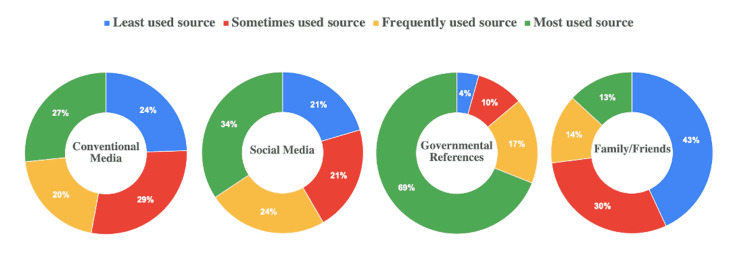
Sources of knowledge

Interestingly, the majority of HCWs (69%) appear to depend upon governmental references as their main source of knowledge, whereas 24% chose social media as the most frequently used source of knowledge. Family/friends were chosen as the least used source of knowledge about COVID-19 (Figure 1).

The proportion of correct to incorrect responses to COVID-19 relevant questions is reported in Table 2. Among the participants, less than half knew the correct incubation period of SARS-CoV-2 (48.91%) while the majority of HCWs (85.4%) chose the correct answers regarding SARS-CoV-2 symptoms. Indeed, over 66% identified the suspected origin of SARS-CoV-2 as it was reported at the time of the study. In addition, the majority of the participants pointed out the correct mode of transmission for the virus as well as the reported health complications of such infection with a percentage of 92.34% and 93.43%, respectively. Similarly, over 70% correctly chose the recommended treatment of choice for COVID-19. Almost all the participants (98.54%) chose the correct measures to reduce the risks of SARS-CoV-2 transmission.

**Table 2:** Perceptions of the participants towards coronavirus (SARS-CoV-2)

Sum Participants (n) = 274	Correct (%)	Wrong (%)
The incubation period of SARS-CoV-2 is 2-14 days	134 (48.91)	140 (51.09)
Skin rash is not a known symptom of SARS-CoV-2	234 (85.40)	40 (14.60)
Bats are thought to be the origin of SARS-CoV-2	181 (66.06)	93 (33.94)
SARS-CoV-2 transmission occurs through direct contact with patients, aerosols, and contaminated surfaces	253 (92.34)	21 (7.66)
Pneumonia, respiratory failure, and death are known complications of SARS-CoV-2	256 (93.43)	18 (6.57)
Supportive care is the current treatment of choice with SARS-CoV-2	201 (73.36)	73 (26.64)
Avoid direct contact with patients, cover mouth and nose, and wash hands to reduce the risk of SARS-CoV-2 transmission	270 (98.54)	4 (1.46)

**Figure 2:**
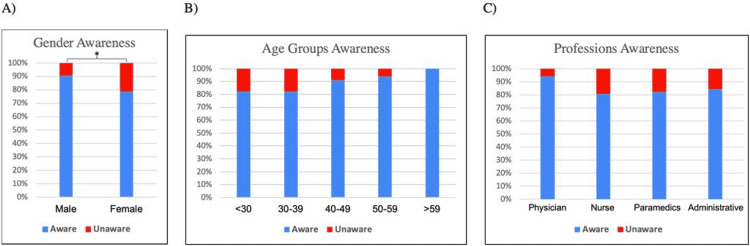
The participants’ overall awareness of coronavirus (SARS-CoV-2) according to demographics and professions Significant association between awareness and the participants’ characteristics is noted by (*) symbol.

The awareness level of the HCWs about COVID-19 is shown in Table 3 and Figure 2 according to their demographics and professions. The male HCWs appeared to have a better level of awareness about COVID-19 compared to the female HCWs (P<0.05). No significant differences were observed among the different age groups or professions in regards to their level of awareness.

**Table 3:** The participants’ overall awareness of coronavirus (SARS-CoV-2) based on their characteristics Values are percentages of the aware and unaware participants of each category. Chi-square P-values of ≤0.05 are marked with a (*) symbol. (f) represents P-value of Fisher exact test.

	%		P	%					P	%				P
	Male	Female		<30	30-39	40-49	50-59	>59		Physician	Nurse	Paramedics	Administrator	
Aware	90	79	0.01*	82	82	91	94	100	0.42^f^	94	81	82	84	0.14
Unaware	10	21		18	18	9	6	0		6	19	18	16	

The HCW's responses to each question in relation to their characteristics are shown in Table 4.

**Table 4:** Association between participants’ characteristics and their perceptions on coronavirus (SARS-CoV-2) Correct and false answer values represent proportional percentages of each category. Chi-square P-values of ≤0.05 are marked with a (*) symbol.

	%		P	%					P	%				P
	Male	Female		<30	30-39	40-49	50-59	>59		Physician	Nurse	Paramedics	Administrator	
The incubation period of SARS-CoV-2 is 2 - 14 days														
Correct	52.38	44.88	0.22	44.78	46.15	60.71	37.50	80	0.15	61.29	41.94	44.05	52.38	0.20
Wrong	47.62	55.12		55.22	53.85	39.29	62.50	20		38.71	58.06	55.95	47.62	
Skin rash is not a known symptom of SARS-CoV-2														
Correct	85.03	85.83	0.85	83.58	86.15	85.71	87.50	80	0.98	83.08	96.77	80.95	82.54	0.07
Wrong	14.97	14.17		16.42	13.85	14.29	12.50	20		16.92	3.23	19.05	17.46	
Bats are thought to be the origin of SARS-CoV-2														
Correct	70.07	61.42	0.13	59.70	62.31	73.21	87.50	100	0.06	81.54	61.29	69.05	50.79	0.02*
Wrong	29.93	38.58		40.30	37.69	26.79	12.50	0		18.46	38.71	30.95	49.21	
SARS-CoV-2 transmission occurs through direct contact with patients, aerosols, and contaminated surfaces														
Correct	91.16	93.70	0.43	94.03	92.31	96.43	81.25	60	0.02*	93.85	95.16	88.10	93.65	0.40
Wrong	8.84	6.30		5.97	4.84	3.57	18.75	40		6.15	4.84	11.90	6.35	
Pneumonia, respiratory failure, and death are known complications of SARS-CoV-2														
Correct	95.24	91.34	0.19	94.03	90.77	98.21	93.75	100	0.41	100	93.55	92.86	87.30	0.05*
Wrong	4.76	8.66		5.97	9.23	1.79	6.25	0		0	6.45	7.14	12.70	
Supportive care is the current treatment of choice with SARS-CoV-2														
Correct	84.35	60.63	0.00*	71.64	74.62	69.64	100	20	0.01*	72.31	51.61	85.71	79.37	0.00*
Wrong	15.65	39.37		28.36	25.38	30.36	0	80		27.69	48.39	14.29	20.53	
Avoid direct contact with patients, cover mouth and nose, and wash hands to reduce the risk of SARS-CoV-2 transmission														
Correct	99.32	97.64	0.25	95.52	99.23	100	100	100	0.21	100	96.77	97.62	100	0.30
Wrong	0.68	2.36		4.48	0.77	0	0	0		0	3.23	2.38	0	

The higher percentage of male HCWs, 50 to 60 years age group, and paramedics (P<0.05) correctly chose the treatment of choice for COVID-19 compared to their counterpart groups. Among the different professions of HCWs, physicians recorded the highest percentage (P ≤0.05) of the correct answers in regards to the origin and the known health complications of SARS-CoV-2 infection. Interestingly, the highest percentage (96.43%) of the correct answers regarding the virus mode of transmission was recorded by the 40-49 years old age group compared to the other age groups. No significant differences were observed between the characteristics of HCWs and their answers to COVID-19 questions.

## Discussion

As the COVID-19 outbreak still spreading across the globe, it is important to assess the awareness and practice of the people towards the infection, especially the HCWs, in order to effectively bring the pandemic to an end. Therefore, we focused in this study on the assessment of the HCWs awareness in one of the major hospitals in Riyadh, Al-Imam Abdulrahman Al Faisal Hospital, which was known to deal with many COVID-19 cases.

Our results have shown that almost all the participating HCWs developed a good knowledge about SARS-CoV-2, although only 70% of them indicated as having the opportunity to participate in an educational session about COVID-19. Interestingly, governmental references were the predominant choice (69%) by the participants as the main source of information regarding COVID-19 whereas the choice of family/friends recorded as the least used source of information. Nevertheless, around one-third of the participants considered conventional and social media as their dependable source of COVID-19 information. Taking into consideration the demographic data of the HCWs, only gender was found to be significantly associated with the overall level of COVID-19 awareness. Nonetheless, upon the analysis of some questions individually for association with the demographic data, a significant association (P<0.05) was observed between the gender, age group, and profession of HCWs and the current treatment of choice for COVID-19. Furthermore, significant (P ≤0.05) associations were observed between the profession of the HCWs and their responses to the origin of SARS-CoV-2 and the known health complications of SARS-CoV-2 infection.

In light of the results of the current study, an overall proper awareness of COVID-19 is evident among the participated HCWs when all the correct responses to the whole questionnaire are considered. To a similar extent, good knowledge is also evident considering the accurate responses about COVID-19 symptoms, mode of transmission, complications, and the currently advised preventive steps. However, some of the HCWs could be considered with an insufficient level of awareness or knowledge as implied by their incorrect responses to some of the COVID-19 relevant questions that may indicate a gap in the health knowledge or practice. Consequently, in a pandemic such as COVID-19, a little gap in the knowledge could lead to serious malpractices and thereby lead to poor infection control that deems all the preventive measures ineffective. Thus, it is of great importance to enforce the key preventive and effective therapeutics measures among the HCWs to break the chain of disease transmission, especially with the uprising cases of COVID-19 all over the world and the occurrence of new variants [19,20].

The incorrect responses on the possibility of SARS-CoV-2 transmission through contaminated surfaces indicate the need for awareness programs that target HCWs, as they are in direct contact with patients and are a high-risk group for the infection and thereby transmission of the disease. Gaps in the HCWs' knowledge about the origin of the virus may indicate the lack of such information at the time of this study. This could be attributed to the uncertain reports about the origin of the virus that was circulating when the study was conducted. Furthermore, the incorrect responses on the incubation period of the virus might be due to the fact that protocols for COVID-19 patients isolation have been changing in accordance with the continuous updates from the WHO and the Saudi ministry of health at the time of the study, thereby, the variation in responses would be expected. Similarly, the variation in the treatment of choice might be due to the reported clinical trials on some drugs claiming to treat COVID-19, such as hydroxychloroquine [21-23].

In this study, nearly 30% of the HCWs never participated in any COVID-19 educational session, and thereby the lack of knowledge in some parts could be explained. However, the reasons behind this phenomenon are yet to be determined. Furthermore, in the context of the current study, few associations were evident between the profession, age, and gender of the HCWs, and some of the COVID-19 questions may indicate the need for further focus on specific demographic characteristics of the HCWs in the future. Our findings strongly suggest that specific demographic characteristics of the HCWs appear to be associated with the level of their awareness of COVID-19, which is consistent with previous observations [10,13].

Other studies from different countries have shown different perceptions of COVID-19. For instance, a study from Egypt reported that risk perception among HCWs was high, with a mean knowledge score of 18.5 out of 24 for 80.4% of the participants [10]. Another study from Cyprus showed that the HCW participants had a positive perception regarding the course of the pandemic, and their knowledge about the coronavirus was satisfactory [15]. A similar study in Jordan revealed that the Jordanian dentists were aware of the COVID-19 mode of transmission, symptoms, infection control, and measures in the dental clinics [24,25]. In contrast, HCWs in the United Arab Emirates showed poor knowledge regarding the transmission and symptoms of COVID-19 [18]. Various sources of knowledge were reported by multiple reports, including WHO and the government websites [25,26], social media [18,27,28], and seminars and workshops [29].

Indeed, the study did have a few limitations including the fact that it was a self-administered questionnaire that was conducted in one center, therefore, it is not representative of the level of awareness of HCWs in Saudi Arabia. Though, it flags the importance of closing the gaps in awareness of COVID-19 among HCWs in order to improve the healthcare services and ensure the safety of the patients. Furthermore, multi-center studies should be included from different regions in Saudi Arabia to evaluate the exact level of awareness among HCWs in Saudi Arabia.

## Conclusions

In conclusion, this study provided an overview about the awareness and knowledge of the HCWs in Al-Imam Abdulrahman Al Faisal Hospital during the COVID-19 pandemic. Though, we cannot generalize the outcome of our study to other medical institutions in Saudi Arabia. Nonetheless, relevant outcomes could also be valid for HCWs in similar settings, thereby highlighting the aspects and need for improvement. In addition, although the main source of information was the governmental or official references, the existence of HCWs who rely on other sources to obtain COVID-19 information, e.g. social networks or family, is indeed alarming as a lot of rumours or fake news circulating among those resources. Thus, the implementation of awareness programs is necessary to prevent such practices and ensure patient safety. Further studies should be warranted in the future to reassess the developments in the aspects of the current study in addition to the HCWs' perceptions towards the licensed COVID-19 vaccines and the national vaccination program.
